# The Temporal Relations of Traumatic Brain Injury, Victimization, Aggression, and Homelessness: A Developmental Trajectory

**DOI:** 10.1089/neur.2020.0050

**Published:** 2021-02-22

**Authors:** Michael D. Cusimano, Melissa B. Korman, Melissa Carpino, Anita Feher, Jeevithaa Puvirajasingam, Stanley Zhang, Stephen W. Hwang, Lorne Tepperman

**Affiliations:** ^1^ Division of Neurosurgery, St. Michael's Hospital, Toronto, Ontario, Canada.; ^2^ Li Ka Shing Knowledge Institute and Keenan Research Centre, St. Michael's Hospital, Toronto, Ontario, Canada.; ^3^ Dalla Lana School of Public Health, University of Toronto, Toronto, Ontario, Canada.; ^4^ Department of Sociology, University of Toronto, Toronto, Ontario, Canada.; ^5^ Centre for Urban Health Solutions, St. Michael's Hospital, Toronto, Ontario, Canada.

**Keywords:** adverse childhood events, concussion, homeless persons, traumatic brain injury, victimization, violence

## Abstract

Traumatic brain injury (TBI) occurs more frequently in homeless persons than the general public. Both homelessness and TBI have been linked to experiences of violence (e.g., aggression and victimization). This study aimed to understand the temporal occurrences of events over the life course that contribute to vulnerabilities to TBI, victimization, aggression, and homelessness. A life-course perspective was used in this thematic analysis of in-person interviews with homeless persons. A total of 33 homeless persons met the inclusion criteria. Twenty-five of 33 (76%) participants had a self-reported history of TBI. Seventy-six percent of TBI events occurred before the onset of homelessness. Assault was the most common mechanism of TBI. During childhood, TBI was a frequently reported event, and parent- or guardian-related physical and sexual abuse were also accentuated with peer abuse, which may have contributed to a unique developmental trajectory. Aggressive behaviors were reported more commonly in persons who previously endured physical, sexual, and emotional victimization early in childhood. The cumulative effect of early adverse events, including TBI and other forms of victimization, subsequent aggression, and further TBI occurring later in life, may create an “at-risk” or vulnerable state preceding homelessness. Precipitating events during adulthood may contribute to a state of homelessness. Homelessness itself may facilitate the context for recurring physical and emotional injury, some of which may be preventable. Future studies should examine the temporality of events related to victimization by physical trauma, such as TBI, aggression, and homelessness.

## Introduction

Traumatic brain injury (TBI) occurs more frequently in homeless persons than the general public. In a Canadian study, almost half of homeless men sampled had suffered a TBI.^1^ Similarly, a UK study reported that approximately half of homeless participants had suffered a TBI, compared to 21% of non-homeless participants,^2^ and were more likely than non-homeless participants to suffer multiple TBIs.^2^ Studies have also shown that the first TBI experienced by many homeless persons pre-dated their onset of homelessness.^3^ However, little is known about the temporal sequences and series of life events that create this vulnerability to TBI and homelessness.

Both TBI and homelessness have been linked to experiences of violence, including aggression and victimization. Irritability and aggression, which are complex constructs with a wide array of biopsychosocial etiologies, have been identified both as an antecedent and a consequence of TBI, with post-TBI aggression occurring in 11–34% of persons.^4–6^ In a study of those who had experienced TBI, the prevalence of aggression (mostly verbal) was 28.4%.^7^ Another study found that TBI patients and their families reported increased irritability, verbal aggression, and annoyance post-injury compared to pre-injury.^5^

TBI is also a complex construct that ranges in severity and can manifest differently based not only on the degree of energy transfer in the inciting event, but also on the patient's cognitive reserve and vulnerabilities in the patient. Clinical experience tells us that a similar blow to the head can have quite different immediate and late sequelae in males and females, across the age spectrum, and depending on whether pre-existing brain pathology (such as past psychiatric illness or cerebral atrophy) exists. For example, a single concussion (a form of mild TBI) uncomplicated by other factors such as psychiatric illness, rarely leads to poor long-term outcomes.^8^ However, TBI has also been linked to experiences of violence,^4^ including aggression in adults and children.^5,6,7,9^ For instance, at-risk youth with untreated TBIs were more likely to behave in a violent manner compared to those who did not suffer a TBI.^10^ A recent study found that both aggression and depression in the early post-TBI period (3 months after injury) were correlated with aggression in the chronic TBI period.^11^ Aggressive behavior post-TBI has been linked to further consequences, including poor social functioning, and is considered a major obstacle for psychosocial recovery.^7^

In addition to its association with TBI, violence has also been widely linked to homelessness. For instance, one study showed that the majority of homeless participants had a history of physical aggression, both before and during periods of homelessness.^12^ Homeless persons of this study were also found to be more susceptible to victimization than members of the general population.^13^ For example, many homeless persons self-report having experienced victimization through physical and sexual assault.^14^ These attacks most often occur in streets or alleys, followed by public parks and homeless shelters,^15^ and often victims report knowing the identity of their attacker.^15^ Victimization preceding homelessness, including physical or sexual victimization in childhood, is also commonly reported by homeless persons.^16^ One study demonstrated that childhood abuse is predictive of future physical victimization and chronic homelessness.^17^ Persons who obtain TBIs because of physical assault by another person are more likely to have poorer psychosocial recovery, and those who experience post-TBI aggression also have delayed recovery.^6,18^ In a Canadian study, assault was the most frequent mechanism of TBI, reported by more than half of the sampled homeless persons.^1^

The literature suggests a clear association between TBI, homelessness, aggression, and victimization; however, detailed information regarding the temporal and developmental trajectories across the life course that contribute to vulnerabilities to TBI, victimization, aggression, and homelessness remains unclear. The life-course perspective (also known as life-course approach) is a multi-disciplinary approach that takes a temporal and societal perspective to the health and well-being of persons, recognizing that early-life experiences can shape health outcomes across an entire lifetime and potentially across generations.^19^ In fact, this approach has been adopted in a variety of settings to disentangle the complex associations between several variables and postulate pathways, linking exposures across the life course to later health outcomes.^20,21^ The aim of the present study was to use a life-course perspective to better understand the temporal occurrences of events related to TBI, victimization, aggression, and homelessness and compare the life trajectories of homeless persons with or without TBI.

## Methods

### Ethics approval

The Research Ethics Board at St. Michael's Hospital (Toronto, Ontario, Canada) approved this study.

### Recruitment, participants, and data collection

Participants were recruited from two homeless shelters (one female and one male) in Toronto, Ontario, Canada. Staff at each shelter informed residents of the study, and posters were displayed in the shelters. Each resident who showed interest was screened using the following inclusion criteria: age 16–70 years, homeless at the time of enrolment, ability to communicate verbally in English, ability to undergo magnetic resonance imaging scanning, and medical stability. Patients with the following comorbidities were excluded: multiple sclerosis, early dementia, Parkinson's disease, neurodegenerative disorders, uncontrolled diabetes, and alcohol-related dementia.

This study used face-to-face semistructured, open-ended interviews that explored developmental trajectories, including relationships with family, parents, caregivers, and peers, education, early adverse childhood events, career, previous TBI, mechanism and timing of previous TBI, mental health status, criminal or delinquent events, past violent episodes, and the use of alcohol and drugs. All interviews were tape-recorded and transcribed.

TBI was self-reported by an intake information questionnaire and by detailed descriptions of events outlined by participants during in-depth interviews. We classified an event as a TBI as long as it matched the Centers for Disease Control and Prevention definition of TBI: “any injury that left someone confused, dazed, or unconscious caused by a bump, blow, jolt or penetrating injury to the head.”^22^ All TBIs were included, regardless of severity. It should be noted that TBI participants experienced a range in severity and number of TBI. Events where a participant explained a scenario that described a TBI without defining it as a TBI (e.g., participant stated being hit on the head and having lost consciousness) were considered “probable TBI.” Events where the participant described a definite hit to the head, but one in which definite loss of consciousness was not described, were considered “possible TBI.”

### Statistical analysis

A life-course perspective^19^ was used in this thematic analysis of in-person interviews with homeless persons to identify significant events and similarities in their lifetime trajectories related to TBI.

NVivo 10 software, specifically the code families and network generation features, was used for interview transcript analysis. The constant comparative method was used,^23–26^ whereby line, sentence, and paragraph segments of transcribed interviews were reviewed and matched to fitting codes. Each transcript was independently analyzed by two members of the research team. To determine consistency of coding, five of the transcripts (representing 15% of the data) were coded independently by both researchers. A kappa score of 86% was obtained, indicating high chance-corrected inter-rater reliability (trustworthiness). The coding process involved the generation of codes which led to categories and, finally, themes that helped formulate a theory. Three coding steps were used in this process, including open coding, axial coding, and selective coding. Any differences in coding were discussed, and the original field notes were reviewed to reach agreement on the most appropriate classification. Codes and categories were adjusted throughout the coding process as themes emerged.

Timelines detailing participants' life course were created and categorized into three epochs: childhood/adolescence (up to and including age 17); adulthood in times specified as not homelessness (when participants did not specify whether events occurred before, after, or during homelessness); and adulthood during the period of homelessness. Both epochs related to adulthood were comprised of events experienced at ≥18 years of age. A specific duration of homelessness was not ascertained. Each person reported experience(s) of homelessness at varying ages and for varying durations of time.

Trustworthiness was addressed in this study in two main ways: 1) the specific grounded theory method that was incorporated in this study helped achieve the Credibility measure by providing “rich” descriptions of the findings, and 2) the rigorous methodological structure and execution of the coding process in NVivo helped with Transferability.

## Results

Forty participants met the inclusion criteria and provided consent. However, 4 participants did not complete the interview and 3 withdrew, resulting in a total of 33 (16 males and 17 females) participants. Of the 33 homeless persons included, 25 had a self-reported history of TBI and 8 had no self-reported history of TBI (Table 1).

**Table 1.**
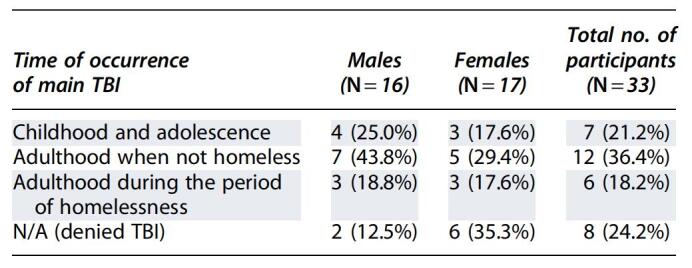
Timing of the Occurrence of TBI in Homeless Persons (*N* = 33)

TBI, traumatic brain injury; N/A, not applicable.

### Time of occurrence of main traumatic brain injury

Of the 25 participants who reported TBI, 19 (76%) indicated this event occurred before homelessness (Table 1).

A number of key nodes coded in the transcripts clearly identified whether events occurred during childhood and adolescence, adulthood in times specified as not homeless, and adulthood during the period of homelessness (see Table 2).

**Table 2.**
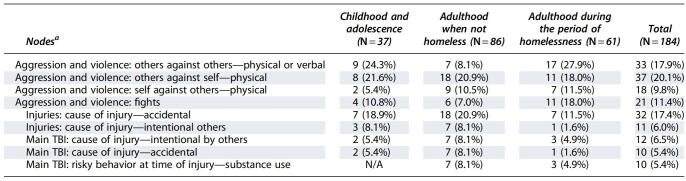
The Number of Relevant Sources (e.g., Participant Transcripts) That Key Nodes Were Coded in during Childhood and Adolescence, Adulthood in Times Specified as Not Homeless, and Adulthood during the Period of Homelessness

^a^ Although there were many more nodes coded in the transcripts, this table depicts all nodes relevant to this article that were present in >20% of transcripts at one of the three time points.

TBI, traumatic brain injury; N/A, not applicable.

### Victimization

Of all 33 participants, 14 (42%) reported experiences of victimization during their lives. The theme of victimization encompassed three subgroups: physical, sexual, and verbal/emotional victimization. Examples of physical, sexual, and verbal/emotional victimization are included in the relevant sections with participant quotes.

#### Physical victimization: traumatic brain injury in childhood/adolescence

Physical victimization was the most commonly reported form of victimization during childhood/adolescence (12 of 33 participants; 36%).

Injuries during childhood were primarily attributable to assault by a family member, most often a parent or guardian. Approximately half of the incidents of assault-related TBI during childhood/adolescence were attributable to reported abuse in the childhood home. A 67-year-old homeless woman with at least two reported TBIs preceding homelessness discussed her experiences with childhood abuse:

“It was when I was about 8 or 9 years old, and my stepmother, uh, hit me on the top of the head with a broomstick…at the time it happened, um, I wanted to drop to my knees.”

Alcohol was often a factor in initiating or worsening the physical victimization. A 61-year-old participant with a history of physical abuse and possible TBI incident said:

“When [my father] became drunk, he immediately came to me and hit me with a strap. Very violent, yeah. I went to high school with 3 black eyes, you know? Over a period of time, there was a lot of physical injury done to me, yeah.”

When physical victimization did not occur in the childhood home, the aggressor was often a child of similar age to the victim. These participants reported revenge, competition in sports, or personal animosities as the reason for the injury. A 47-year-old male with a history of substance abuse and who experienced a possible TBI in childhood explained that incidents with peers were provoked by *“someone call[ing] your mom a name, or you a name. You know, stuff like that.”*

#### Physical victimization: traumatic brain injury in adulthood

Victimization also occurred during the period of adulthood before homelessness. Again, physical victimization emerged as the most common form, reported by the majority of participants. The majority of participants were physically victimized by strangers, in fights, robberies, or mistaken/“accidental” encounters. These types of encounters included situations where the participant accidentally bumped into someone who then retaliated, or cases of miscommunication where the assaulter mistakenly thought that the participant had performed some transgression. A 54-year-old homeless participant who was hospitalized for a TBI after being assaulted by a stranger during early adulthood said:

“I bumped into this individual, who just wasn't confident in what I was up to. And he just punched me in the face and I fell backwards and struck my head on the ground.”

A large number of victimized participants also reported situations of domestic or partner assault, which was generally attributable to jealous or controlling partners. A 51-year-old woman with a history of physical and sexual abuse, as well as substance abuse, said:

“I was in a relationship with a man that was very abusive. He drugged me and he, um, he raped me and he abused me, and he beat me…I've found myself in this position so many times, and cause there have been other men that have gotten close to abusing me that I've sort of seen them, seen the bells and the whistles and gotten out in time, but I seem to invite these, these abusive types into my life.”

Fewer participants reported victimization during the period of homelessness than during periods when they were not homeless. In fact, less than half of the participants indicated any form of victimization during the period of homelessness; however, over half of those who reported victimization during the period of homelessness suffered from physical victimization. Similar to adulthood before homelessness, the assailant was most commonly a stranger. One person of 33 (3%) reported intimate partner violence as the cause of their TBI during the period of homelessness.

Most TBIs acquired during the period of homelessness were reported to be financially motivated, often occurring during either a robbery or a fight over money. In multiple cases, the assaulter approached the participant with the intention to rob him or her, and the participant sustained a TBI during the encounter. A 39-year-old immigrant woman who ran away from home as a teenager explained her TBI as such:

“*She was trying to take the money and we got into a big brawl and we got into a big fight and we started fighting when she was trying to attack me, trying to take the money from me…so I was fighting her, like just fighting her, and she hit me in the head a couple of times with her fist…”*

#### Physical victimization over the life course

Assault was the most common mechanism of TBI, with more than half of the participants indicating one assault-related TBI during their lifetime. A similar number of homeless persons suffered an assault-related TBI at each stage of life (childhood/adolescence, adulthood in times specified as not homeless, and adulthood during the period of homelessness). The majority of participants experienced an assault-related TBI before their first episode of homelessness.

#### Sexual victimization: childhood/adolescence

Reported by 8 participants (24%), sexual abuse was the second-highest form of victimization during childhood/adolescence. Perpetrators of sexual abuse were commonly persons in positions of authority, such as teachers or leaders of an institution. A 55-year-old man who experienced physical and sexual abuse during childhood said:

“I got sexually abused there by the Catholic priest.”

In some cases, the abuser was a family member, neighbor, or friend of a family member (e.g., their parent's significant other). A 67-year-old woman who was sexually assaulted by her stepbrothers and physically abused by her stepmother during childhood said:

“and the two older sons, the two are the ones that sexually assaulted me, starting at two years old.”

Other cases of sexual abuse during childhood seemed to be based on convenience for the abuser(s) and vulnerability of the victim. A 52-year-old woman who lived in a children's home as a child and reported no history of TBI said:

*“Yeah and I didn't have anywhere to sleep and um, some guys told me that I could sleep in the graveyard eh? And they raped me.*”

#### Sexual victimization: adulthood

Sexual victimization during adulthood was less commonly reported (*N* = 2; 6%). In each case, the perpetrator was a friend or intimate partner, and only 1 participant reported sexual victimization during the period of homelessness.

#### Verbal/emotional victimization: childhood/adolescence

Just less than one quarter of participants reported verbal/emotional victimization during childhood, fewer than either sexual or physical victimization. In all reported cases, the perpetrator was a family member, often an abusive parent threatening the participant or making them feel diminished in worth. A 51-year-old woman who experienced physical, emotional, and sexual abuse during childhood said:

“My father…he didn't beat us or anything, but uh, you know, he threatened to come and shoot us all with a sawed off shotgun. He was very violent and…very frightening, so there's a lot of fear.”

#### Verbal/emotional victimization: adulthood

During adulthood before the first experience of homelessness, one quarter of participants experienced verbal/emotional victimization. The abuse came from various outlets, including abusive domestic situations, authority figures like landlords and managers, and strangers on the streets. A 31-year-old transgender woman who moved into a women's residence to get away from her boyfriend said:

“I broke up with my boyfriend…because he…had a drinking problem and…he would get very verbally abusive and…I gave as many chances as I could but I'm just at the end of my rope.”

Similarly, 8 participants (24%) spoke of verbal/emotional victimization during the period of homelessness. A 67-year-old male with no reported history of TBI said:

“Just people talk to you and they assume what kind of person that you are and that stuff. They say things that are condescending or rude…If I wore a tie and…could look like I was on Bay St., people would talk to me the same way, you know?”

### Aggression

For the purposes of this study, aggression was defined as circumstances where *the participant* was violent toward another person or got into a fight. This is in contrast to the theme of victimization where the participant was victimized by another person, and in this theme, the participant committed some violent act on someone else. The most common type of aggressive act across all life stages was engaging in a physical altercation with another person. A 44-year-old male with a history of violent behavior and emotional trauma, and a probable TBI in childhood, said:

“I got in a fight with two dudes — they bottled me here and stabbed me in this arm.”

#### Trajectory of aggression

In contrast to TBI and physical and sexual victimization, which primarily occurred during childhood or adolescence, self-reported aggressive behaviors primarily occurred during adulthood before homelessness. Aggressive behaviors were reported more commonly in persons who previously endured physical, sexual, and emotional victimization early in childhood. Only around one quarter of all participants engaged in an aggressive act during childhood/adolescence. In some circumstances, these aggressive acts were seen as a form of defensive behavior meant to protect one's self and purely as aggression in others. During adulthood, but before homelessness, around half of participants committed aggressive acts. During the period of homelessness, approximately one third of the participants committed violent acts against others. Self-reported physical aggression toward another person often occurred in response to verbal provocation. For instance, a 67-year-old male with multiple convictions said:

“I knocked a guy out a few weeks ago for calling me a n****”

Participants also spoke of instigating aggressive behaviors and physical altercations. They described putting themselves into potentially high-risk situations where they acted aggressively rather than avoiding them. In some cases, it was in order to protect their belongings from people known by the aggressor. For instance, a 51-year-old woman with a history of childhood physical and sexual abuse, as well as drug and alcohol abuse, said:

“I drove up on them actually as they were robbing the house. And I went ballistic; I ran in, grabbed a knife and ran in the house looking for blood!”

However, in other cases, the participant was completely unrelated to the person. A 44-year-old male with a history of violent behavior said:

“I caught a dude robbing [name] and confronted him about it and he pulled a knife on me. I kicked the knife out of his hand, threw my coffee on his face, left him crying like a baby on the floor…”

**FIG. 1.**
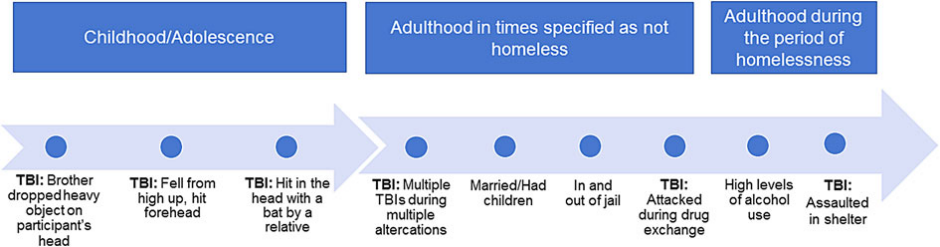
Life trajectory of a participant who had multiple TBIs, was physically victimized as a child, showed aggression in adulthood before homelessness, and increased alcohol intake during homelessness. TBI, traumatic brain injury.

A trajectory highlighting the repeated victimizations and subsequent aggressions experienced by this participant is outlined in Figure 1.

#### Childhood traumatic brain injury and later aggression

Participants who suffered a TBI in childhood were compared to those who did not suffer a childhood TBI. Among persons who experienced a TBI during childhood (*N* = 7), 72% reported being aggressive in adulthood before homelessness, whereas just under 40% indicated aggression during the period of homelessness.

For persons with no reported TBI during childhood (*N* = 8), 25% reported aggressive behavior during adulthood before homelessness and also during the period of homelessness. Although these participants had no reported TBI in childhood, they all reported adverse childhood experiences during childhood. Interestingly, more than half of the participants with no reported childhood TBI also reported no aggressive behavior.

#### Childhood victimization and later aggression

Participants who experienced physical, sexual, and verbal/emotional victimization during childhood were compared to those who did not. Among those who were victimized during childhood, more than half reported being aggressive in adulthood before homelessness, but less than one quarter during the period of homelessness. A 51-year-old man who witnessed his father beat people up when drunk said:

“I used to be really violent back then and now I don't do it anymore…I don't know, somehow when my head injury…when I woke up in hospital…somehow I didn't…I didn't wanna hit nobody. I didn't want to hurt nobody.”

Among those who did not experience childhood victimization, few reported aggressive behavior during adulthood before periods of homelessness. However, this number was slightly higher during the period of homelessness, with 1 additional participant reporting this behavior.

### Location of victimization

During childhood, victimization largely occurred in the childhood or adolescence home setting. During adulthood before homelessness, around half of the physical victimization (assaults) by strangers occurred in places that may be considered risky environments (e.g., bars, alleyways, strip clubs, at parties, and on the streets). Some factors that were common in these environments were lack of bystanders, presence of intoxicated persons, and a lack of privacy. A 47-year-old male who was assaulted by a group of men during early adulthood said:

“I was walking in an alleyway and three guys jumped me, beat me up. They busted up my leg and one of them hit me in the head with a stick.”

During periods of homelessness, the most frequently cited location of physical victimization was the homeless shelter rather than the street. Nearly half of those who reported victimization indicated that these experiences took place at the homeless shelter in which they were staying. Further, approximately one third of reported assault-related TBIs during the period of homelessness occurred in the homeless shelters in which the participants were residing. In addition, roughly one third of assault-related TBIs occurred in other types of risky environments, and the rest did not discuss where the incident took place. These findings are well demonstrated in the trajectory of the participant outlined in Figure 1.

## Discussion

This study examined assault-related TBI, victimization, and aggression among a sample of homeless participants, the majority of whom had at least one TBI over the life course. Previous studies found that the first TBI suffered by homeless persons often occurred before the onset of homelessness.^2,3,27,28^ The current study replicated this, given that it revealed that TBI was a frequently reported event during childhood and fewer assault-related TBIs were reported during the period of homelessness than at other times. The same pattern was found for victimization and aggression. Both themes were also frequently reported during adulthood before homelessness. Although we found fewer of these events during the period of homelessness, confounding factors such as duration of homelessness were not recorded and may have been relatively short compared to the periods of time where persons were not homeless.

Our study found that TBI was a frequently reported event during childhood and many of these injuries occurred in the setting of victimization, including sexual abuse, often during childhood. During childhood, early events of guardian-related physical and sexual abuse were also accentuated with peer abuse. These forms of victimization may have contributed to a unique developmental trajectory within these persons, characterized by childhood adversity, followed by aggression and victimization in adulthood, and followed by homelessness.

Assault-related TBIs were the most common type of brain injury reported by this population, as consistent with other studies.^1^ As shown by previous studies, TBIs increase the risk of poor social integration and unemployment.^18,29^ Previous studies have focused on guardian abuse as a common reason for assault-related TBIs in childhood.^30^ In addition to our study supporting these previous findings of early-life injuries, our results also emphasize that fights and competition with other children is another plausible cause of childhood TBI. This highlights the importance of focusing attention on preventative methods broadly to protect children in school and extracurricular settings, such as sports, not just in the home.

Another important finding regarding assault-related TBIs in this sample was that in adulthood, both before and during the period of homelessness, most assault-related TBIs were caused by strangers to the victim. Contrary to earlier work,^13^ we found that most injuries in childhood were perpetrated by aggressors that the child knew. We posit that persons who experienced childhood abuse, and the multitude of factors associated with it, may have embarked on a developmental trajectory characterized and shaped by these early adverse events and environments inflicted upon them by persons they knew.

In some participants, repeated TBI continued to occur during the period of homelessness. It is important to note that approximately one third of reported assault-related TBIs during the period of homelessness occurred in the shelter environment, with many of the assaults being financially motivated. These findings suggest that the number of TBI events during periods of homelessness could potentially be reduced if homeless persons had a safe place to spend their time or lock up their belongings, given that it would remove the reasoning behind most assaults that reportedly occurred in the shelter. This creates a potential opportunity for preventive strategies, such as those aimed at improving staff awareness, and procedures to reduce motivations for violence in the shelter environment, such as means to safeguard belongings and money.

Participants were also almost equally likely to report emotional/verbal victimization as physical victimization during homelessness, demonstrating that emotional (not just physical) safety should also be a priority for programs that seek to improve the circumstances of homeless persons. Given that around half of victimized participants reported being victimized within the shelter, there is a need to improve conditions within homeless shelters and take measures to increase safety.

Both victimization and aggression themes were frequently reported during adulthood before homelessness, and continued, albeit at lower rates and in different forms, during the period of homelessness. In our view, this aggression may be a reaction to victimization to a variety of insults, including TBI, but could also be a survival behavior in a harsh setting. Further, our study showed the temporal sequences of early forms of victimization to later aggression and continued cycles of victimization. Although earlier studies have found that post-TBI aggression occurs in 11–34% of persons,^4,6^ and verbal aggression toward others is a frequent manifestation of this,^7^ participants in our study demonstrated repeated cycles of victimization related to these aggressive behaviors from others. Given recent findings that both aggression and depression in the early post-TBI period (3 months after injury) are correlated with aggression in the chronic TBI period,^11^ it is not surprising that participants in this study also showed continued cycles of victimization, which included repeated TBI into adulthood before and during the period of homelessness.

Our results support the finding that aggressive behavior after TBI has been linked to further consequences, including poor social functioning, and is considered a major obstacle for psychosocial recovery.^7^ Our results, which show a temporal relationship between early victimization and later cycles of aggression and revictimization, suggest that participants' state of social functioning may have been initiated by an early developmental trajectory that included early childhood physical, mental, and sexual abuse. Our results raise the possibility that this may also create an at-risk or vulnerable state that may be associated with homelessness.

Given these findings, the results of our study may posit an association between early victimization (encompassing TBI as well as physical and sexual violence) and later-life aggression and violence and is supported by earlier work.^10,31^ When comparing persons who were victimized in childhood/adolescence with those who were not, our study found that whereas a high number of those victimized during childhood/adolescence reported aggression in adulthood, that number was lower during the period of homelessness. A much smaller percentage of those not victimized in childhood/adolescence reported being aggressive in adulthood; however, a significant proportion of these participants continued their pattern of aggression into homelessness. Although our work and that of others supports the idea that childhood victimization is likely associated with an increased likelihood of aggression in later periods of life,^32^ a causal association between early victimization (including TBI) and aggression later in life, and whether this aggression is less apparent during adulthood homelessness, cannot be inferred because of the qualitative nature of our study, small sample size, and because a number of confounding factors, such as the duration of homelessness and non-homelessness, were not controlled for in the present study.

We believe that it is entirely plausible that TBI may cause aggression and aggression may cause TBI, and both may be related to a third common factor such as adverse childhood experiences. Future studies should further examine the interesting relationships we have found, and whether a cause-and-effect relationship between victimization by physical trauma, such as TBI, other forms of trauma and neglect, and aggression/violence, exists.

During adulthood, both before and during the period of homelessness, participants recounted repeated assault, often precipitated by aggressive tendencies manifested within physical environments of risk, which included the homeless shelter. The fact that these events were inflicted by strangers within these environments, often driven by altercations related to money, provides a potential avenue for prevention strategies. Homeless shelter staff, although likely aware of these altercations, may benefit from education regarding the behavioral and past history context of these persons and could play an important role in the prevention of future TBI and adverse events.

**FIG. 2.**
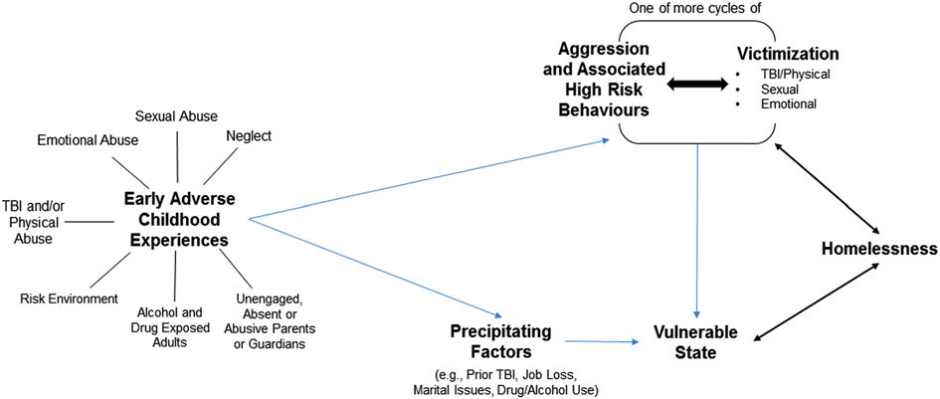
Proposed web of association between early adverse childhood experiences/influences (including TBI), cycles of aggression and victimization, and homelessness. Early adverse childhood experiences are shown to be associated with violent behaviors or other precipitating factors, which may make a person vulnerable to homelessness or TBI. Homelessness itself may create a vulnerable state and/or increase the likelihood of aggressive behaviors or victimization. TBI, traumatic brain injury.

When considered in relation to other work in this field, our findings suggest a model linking cycles of different forms of victimization, including TBI (often beginning early in life), which may characterize specific developmental trajectories at risk for adverse future social and health behavior (see Fig. 2). These results suggest that these early-life events often occur in a context, though not necessarily only within a context of alcohol and drug use, among important adults related to the child. We posit that later behaviors like aggression, recklessness, and poor insight about behavior on future consequences may manifest and create susceptibility for further cycles of victimization, including further TBI, and one outcome of these may be homelessness. This model would imply that alcohol and drug use and other risk behaviors may become relatively prevalent and are associated with further cycles, continuing into adulthood. These experiences may create a vulnerable state that may, in some, lead to homelessness by a variety of precipitating factors, such as job loss, marital breakdown, drug and alcohol use, etc. (Fig. 2).

This model might also be useful to contextualize our understanding of other adverse events such as mental health outcomes, addictions, and criminality. Indeed, our conceptual model provides a further explanation of the findings of previous research, which observed an association between victimization and an increased likelihood of aggression in later periods of life, and provided evidence of a trend between brain injury and later aggression.^32^ Interestingly, this work may have a biological basis as demonstrated by animal models of early-life stresses that link hyperconnectivity in frontolimbic circuits and increased anxiety.^33^

### Limitations

Data reported in this study were based on self-report, rather than on medical records. Self-reported measures were used because medical records often did not exist, given the context of and date when events occurred. Physical or sexual assault of children by known adults is rarely reported to authorities. As well, by using self-report, we aimed to ensure that participants' views were represented in order to provide a more complete life-course perspective. However, given that many participants suffered cognitive issues attributable to their brain injuries, drug or alcohol problems, or may have simply forgotten, misspoke, or left out details, some of the data reported may not represent the truth or be affected by recall bias. However, participants would have little motivation to embellish their stories given that the research team had no relation to shelter staff and were neither their healthcare providers nor linked to their financial or other status in any way. Further, recent work from Norway has shown that only a minor proportion of the association between childhood abuse and chronic conditions was driven by recall bias attributable to the respondent's mental health, and the associations between self-reported childhood disadvantage and health outcomes in adulthood are not driven by respondents' current psychological state.^34^

Finally, our sample size may not represent the realities of a wider range of homeless participants; however, our sample was equally balanced with males and females, and our findings were consistent and repeatedly demonstrated by participants. Future studies should include a larger sample, with more directed questions that focus on both experiences of TBI and violence, confirmed, if possible, by medical reports. All the frequencies in the present article are merely based on what was reported, and may change in future studies according to the selected sample and the comprehensiveness of questions given to participants. Therefore, it is not the actual frequencies reported that should be the focus of the implications of our work, but the fact that certain events or surrounding circumstances were reported at various stages in the life course of these persons.

Given that this study was conducted using a constant comparative approach with thematic analysis to develop conceptualizations of the roles of TBI and other life events in the lives of homeless persons, certain events were reported in an estimated time frame based on the surrounding context of the interview transcript. This study should set a precedent for future studies to adopt a timeline format, given that it allowed for an individualistic approach to the issue, while revealing patterns of change over the life course. Finally, our work was limited to a single geographical location in one of the world's most multi-cultural cities. Future work should replicate this work in other geographical and cultural settings to assess the generalizability of our findings.

### Implications

The results of this study highlight the need for prevention measures and strategies to decrease adverse childhood experiences, such as physical and sexual victimization, as well as the high prevalence of TBI and violence, in persons susceptible to future homelessness or who are presently experiencing homelessness. The results from this life-course perspective emphasize that prevention measures aimed at all life stages, starting importantly with healthy childhoods, could potentially hold promise for reducing victimization and potential adverse outcomes like homelessness. Immediate strategies for homeless persons to safeguard possessions during the homeless state could have direct impacts given the high frequency of financially motivated stranger assaults. As well, given that a high number of assault-related TBIs (and other forms of victimization) occurred within homeless shelters themselves, improving education of staff and safety standards in shelters should be prioritized.

## Conclusion

The cumulative effect of early adverse events, including TBI and other forms of victimization, subsequent aggression, and further TBI that occurs later in life, may create an at-risk or vulnerable state before homelessness. Precipitating events during adulthood may contribute to a state of homelessness. Homelessness itself may create the context for recurring physical and emotional injury (often related to financial/environmental factors), some of which may be preventable. Future studies should examine the temporality of events related to victimization by physical trauma, such as TBI, aggression, and homelessness. This work, and the model leading from it, highlights the need and potential benefit of adopting a life-course approach to promote healthy childhoods, including the implementation of strategies that aim to prevent TBI, physical and sexual abuse, and aim to create safe environments for homeless adults. Finally, this work has implications for strategies aimed at the development of social environments that promote healthy childhood and later-life environments, strategies that aim to lessen the risks of those vulnerable to homelessness, as well as policies and programs that aim to support the homeless, especially those with previous TBI.

## Abbreviation Used

TBI: traumatic brain injury
